# Alterations in gut microbial metabolic pathways following bariatric surgery assessed by 16S rRNA gene sequencing

**DOI:** 10.1017/gmb.2026.10017

**Published:** 2026-01-19

**Authors:** Nisreen Rashad Tashkandy

**Affiliations:** Department of Biological Sciences, King Abdulaziz University, Saudi Arabia

**Keywords:** gut microbiome, overweight, iron supplementation, metabolic pathway

## Abstract

Researchers have studied gut microbiota changes following bariatric surgery (BS), but not gut diversity and function in patients who fail to reduce weight. Stool samples were collected from three groups of women: 15 women who did not lose weight after BS (“Yes” group), 9 overweight women without surgery, and 8 slim women (“No” group). 16S ribosomal RNA gene sequencing and PICRUSt2 were used for the analysis. The surgery and control groups had equal alpha and beta diversity, perhaps due to the high proportion of overweight participants (n = 24). All groupings were dominated by Bacteroidota and Bacillota. *Barnesiellaceae* decreased with BS, although *Streptococcaceae* remained frequent in overweight people. The iron supplementation group had High abundance of *Atopobiaceae* and *Prevotellaceae*. *Barnesiellaceae* abundance was considerably lower in both surgical groups (with and without iron supplementation) than in the no-iron and no-surgery groups. The ornithine degradation and haem biosynthesis routes use different metabolites than the glycine super system. Finally, the “Yes” group significantly upregulated PWY0–1241, PWY-5177, and PWY-5855 signaling pathways. In conclusion, gut bacteria and metabolic functions may predict weight loss after surgery better than diversity markers. The requirement for orthogonal validation assays is suggested by pathway analysis outperforming diversity metrics.

## Introduction

Obesity represents a significant global health challenge that extends beyond its direct metabolic effects to influence the composition and function of the gut microbiota. Research has consistently demonstrated that obesity alters gut microbial communities by changing their composition and reducing overall microbial diversity (Turnbaugh et al., 2009). These alterations may contribute to the pathophysiology of obesity and its associated metabolic disorders, although the precise mechanisms remain an active area of research. Bariatric surgery (BS) has emerged as one of the most effective interventions for severe obesity, not only facilitating substantial weight loss but also improving metabolic parameters and comorbidities. Interestingly, these beneficial effects often precede significant weight reduction, suggesting mechanisms beyond simple caloric restriction (Ilhan et al., 2020). However, more than half of the patients (53.3%) experienced significant weight regain at 6 years of follow-up, and a pattern of weight regain was observed regardless of the surgical procedure. Therefore, the major challenge to BS success is maintaining long-term weight loss (Alfadda et al., 2021).

The human gut microbiome is primarily composed of two types of bacteria – Bacillota and Bacteroidota – which together account for more than 90% of the bacteria in the intestines. The relative abundance of these phyla, often reported as the Bacillota/Bacteroidota ratio, has been proposed as a key marker of the gut microbial composition and metabolic health (Paziewska et al., 2024; Zhang et al., 2020). However, the literature presents contradictory findings regarding this ratio in individuals with obesity. While earlier studies suggested that obesity is correlated with an increased Bacillota/Bacteroidota ratio, more recent investigations have challenged this paradigm. For instance, Lau et al. (2021) did not observe significant alterations in this ratio in obese subjects, whereas Georgiou et al. (2022) reported the opposite trend, with an increase in the abundance of Bacteroidota and a decrease in the abundance of Bacillota in obese individuals. These contradictory findings highlight the complexity of gut microbiota dynamics and suggest that simplistic ratios may not fully capture the metabolic implications of changes in the microbial community composition. This inconsistency in the literature is more nuanced in understanding the role of the gut microbiota in obesity and its treatments.

Accumulating evidence indicates that BS significantly alters the gut microbiome, potentially contributing to its metabolic benefits. These microbial alterations appear to vary with the specific surgical procedure employed, with studies generally reporting increased abundances of Bacteroidota and Proteobacteria following BS (Alqahtani et al., 2023). However, disentangling the particular contributions of reduced caloric intake, altered food quality, and the surgical procedure itself to these microbial changes remains challenging (Ciobârcă et al., 2020). The long-term stability of microbial changes induced by BS also warrants further investigation. A systematic review (Mohammadzadeh et al., 2025) highlighted significant knowledge gaps regarding the persistence of these alterations and their long-term effects on metabolic health. Longitudinal studies are needed to determine whether these changes persist and how they influence metabolic outcomes over time. Of particular relevance to clinical practice is the observation that not all patients achieve comparable weight loss outcomes following BS. A cross-sectional study (Gutiérrez-Repiso et al., 2019) examined gut microbiome profiles in patients with different weight loss outcomes after gastric bypass (success, primary failure, and weight regain). The findings revealed that patients who experienced primary failure exhibited distinct gut microbiota patterns and poorer metabolic health than patients who did not experience primary failure, suggesting a potential link between the gut microbial composition and BS outcomes. A significant but often overlooked complication following BS is the development of nutritional deficiencies, particularly iron deficiency. Assakran et al. (2023) reported a high incidence of anaemia risk factors in women following BS, a finding with important implications for postsurgical care. Iron deficiency commonly occurs in patients with BS because of reduced gastric acid secretion, which impairs iron absorption (Engebretsen et al., 2018). Consequently, iron supplements are frequently prescribed to these patients. However, this therapeutic approach introduces another layer of complexity to the gut microbiota equation, as iron availability significantly influences the microbial community structure and function. Iron supplementation can substantially change the gut microbiome, possibly leading to more harmful bacteria in the digestive system. Studies have demonstrated that oral iron supplementation may reduce the abundance of beneficial bacteria, such as *Lactobacillus* and *Bifidobacterium*, while promoting the growth of potentially detrimental bacteria, including *Bacteroides* and *Clostridium* species. These alterations can lead to gastrointestinal symptoms such as diarrhoea and may influence the overall metabolic effects of BS. The effect appears to be dose-dependent, with higher iron intake associated with more pronounced changes in the microbial composition. For example, Shearer et al. (2024) found that high iron intake increased the abundance of *Proteobacteria* and decreased the abundance of beneficial gut microbes such as *Akkermansia* and *Butyricicoccus.* Furthermore, experimental studies in mice have shown that various routes of iron administration – oral, intravenous, or chronic transfusions – all alter the gut microbiota composition, suggesting that iron itself, regardless of the administration method, is a key factor driving these changes (La Carpia et al., 2019).

Given the complex interplay between BS, weight loss outcomes, and iron supplementation in exerting effects on the gut microbiota, this study aims to investigate whether the gut microbiota composition differs between individuals who are overweight and those who have undergone BS and those who are overweight but have not undergone surgery. Of particular interest are those patients with BS who failed to achieve the desired weight loss, as understanding their gut microbial profiles may provide insights into the biological factors underlying treatment resistance. Additionally, we sought to examine how iron supplementation, which is typically prescribed after BS, influences gut microbial communities and whether these effects interact with surgical outcomes.

## Materials and methods

### Study population

Thirty-two faecal samples were obtained from female volunteers, with an average age of 51 years, to evaluate the gut microbiome. Fifteen postsurgical overweight individuals, nine overweight individuals, and eight normal-weight volunteers were included in this study, and a comprehensive summary of all the phenotypes of each participant is provided in Supplementary File S1. The inclusion criterion was females who underwent BS surgery and who failed to achieve the desired weight loss. Females who had received antibiotic therapy 3 months before sampling, as well as individuals with diarrhoea during the same interval, were excluded from this study. Details about the study population are presented in Table 1. This study received approval from the King Abdulaziz University Unit of Biomedical Ethics Research Ethics Committee with the NCBE Registration No. HA-02-J-008.

**Table 1.** Demographic and clinical characteristics of the women based on their weight and history of bariatric surgery

Variables	Bariatric surgery *n* = 15	Overweight, no surgery *n* = 9	Normal weight, no surgery *n* = 8
Age, years	35–70	40–75	40–70
CODE	P1 to p15	C1, C2, C3, C4, C5, C10, C12, C14, C15	C6, C7, C8, C9, C11, C13, C16, C17
Postmenopausal Menstruating Premenopausal	2 9 4	4 2 3	4 2 2
BMI, kg/m^2^	25–43	26–44	20–23
Duration after surgery	1–20 years		
Comorbidities Diarrhoea Constipation Cholecystectomy	8 4 2	0 5 0	0 0 0
Iron supplementation	11	0	0

### Microbiota sequencing and taxonomic assignment

Total faecal DNA was extracted using the QIAamp PowerFecal Pro DNA Kit (QIAGEN, Hilden, Germany) according to the manufacturer’s protocol. The DNA concentration was measured using gel electrophoresis and a NanoDrop-1000 spectrophotometer manufactured by Thermo Fisher Scientific (formerly Nanodrop Technologies) in Wilmington, DE, USA. The concentrations of the DNA samples ranged from 64.113 to 465.63 ng/μL. The samples were stored at −20 °C before sequencing. The V3 and V4 regions of 16S rRNA were amplified using the primers 341F (CCTAYGGGRBGCASCAG) and 806R (GGACTACNNGGGTATCTAAT) with barcodes. All polymerase chain reactions (PCRs) were performed with Phusion® High-Fidelity PCR Master Mix (New England Biolabs) conducted by Novogene Bioinformatics Technology Co., Ltd. The amplicons were sequenced on an Illumina paired-end (PE) platform to generate 250 bp raw PE reads, which were merged and pretreated to obtain clean tags. Sequencing of the 32 samples yielded, on average, 197,684 ± 5,627 (Standard Error of the Mean - SEM) raw reads. The raw sequence data have been submitted to the GenBank Sequence Read Archive under Submission ID: SUB14906866, BioProject ID: PRJNA1193787. The chimeric sequences in the clean tags were detected and removed to obtain the effective tags that could be used for subsequent analysis. After quality control and chimeric sequence removal, 169,427 ± 5,412 (SEM) reads with an average length of 415 ± 0.63 (SEM) base pairs remained per sample. FASTQ files were transformed into a sample-by-sequence table using the DADA2 R package (v.1.32.0) (Callahan et al., 2016). At the genus level, taxa were assigned using the assignTaxonomy() function, whereas species were assigned to sequences with the “add Species” function of the same package. DADA2-formatted SILVA database (v. 138.2) files retrieved from https://doi.org/10.5281/zenodo.14169026 were used as references in both cases (Klindworth et al., 2013).

### Statistical analysis

Within-sample (alpha) diversity was assessed using the observed taxa and Shannon index as calculated using the estimate_richness function of the phyloseq R package (v. 1.48.0) (McMurdie and Holmes, 2013) and compared between groups with the Wilcoxon rank-sum test (for two conditions) or Kruskal–Wallis and Dunn’s post hoc tests (for multiple conditions) (Costello et al., 2009; Turnbaugh et al., 2010). Across-sample (beta) diversity measurements were performed to examine sample dissimilarity using the Aitchison distance, as described elsewhere (Gloor et al., 2017), as it is an appropriate method that considers the compositional nature of 16S rRNA sequencing data. Permutational multivariate analysis of variance (PERMANOVA) was performed using the *adonis* function from the *vegan* R package. A permutation test for constrained correspondence analysis from the same package (*permutest* function) was used to examine the homogeneity of multivariate dispersions (*betadisper*). rrnDB data (v.5.7) (https://rrndb.umms.med.umich.edu/downloads/) were used to correct for differences in gene copy numbers (GCNs) before any downstream analyses (Stoddard et al., 2015). More specifically, the genus-level Operational Taxonomic Unit (OTU) data were divided by the respective GCN. Taxa not recorded in the database were excluded from downstream analyses. Relative abundance was compared between taxa classified in each group using either the Wilcoxon rank-sum (two conditions) or Kruskal–Wallis *H* test, followed by the Dunn post hoc test (multiple conditions), as implemented in the vanilla R function. Statistically significant differences in relative abundance were defined by an adjusted *p*-value < 0.05.

### Estimating the functional metagenome

The functional high-throughput 16S rRNA gene was predicted using Phylogenetic Investigation of Communities by Reconstruction of Unobserved States 2 (PICRUSt2) (V 2.3.0) (Douglas et al., 2020). Functional predictions were conducted using 16S sequencing data derived from the Amplicon Sequence Variants, the Kyoto Encyclopedia of Genes and Genomes (KEGG), and the MetaCyc pathway databases. The ggpicrust2 R package (Yang et al., 2023) was employed to visualize and analyse the abundance of the predicted pathways.

## Results

**Figure 1.**
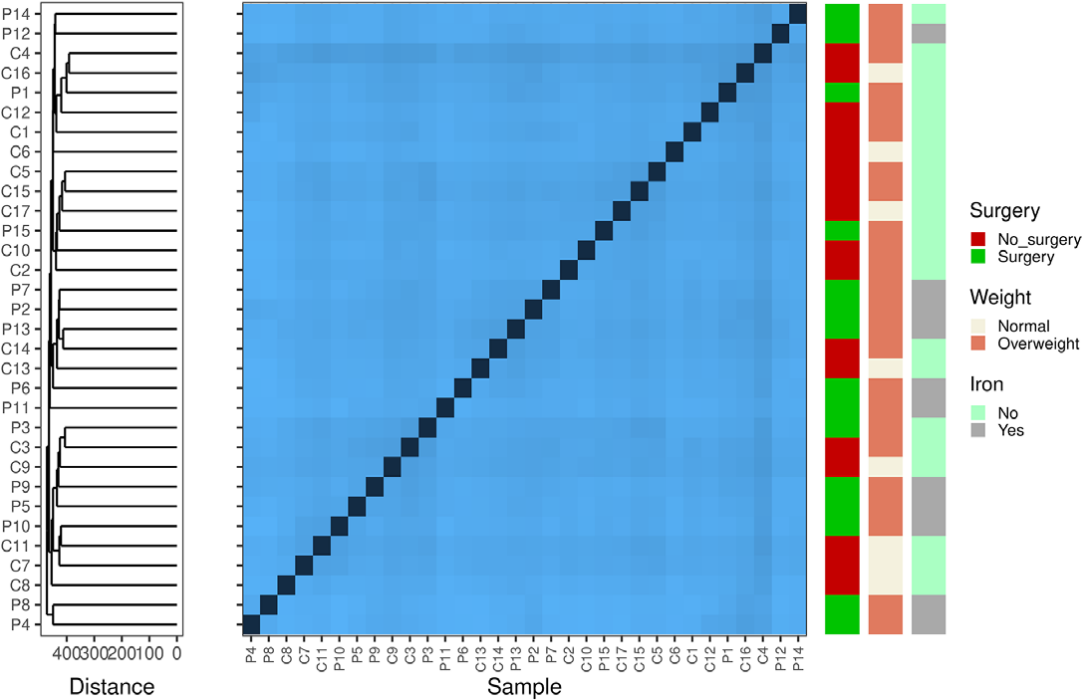
Beta diversity-based Aitchison distance clustering reveals potentially interesting groups among samples. The heatmap shows the pairwise Aitchison distances (centred log-ratio transformed) between samples. These differences indicate that the microbial composition of the cohort differed. Darker colours indicate closely related samples. On the left, hierarchical clustering with complete linkage groups the samples based on their beta diversity profiles. The arrangement of the clusters suggests no important connection between the microbial profiles and clinical factors, such as surgery, weight, and iron levels.

First, we aimed to examine within-sample diversity changes driven by surgery, differences in weight, and iron supplementation (Figure 1). Surgery and iron intake were largely confounded (Table 1) and were thus examined in a 2 × 2 design. Comparisons of the Shannon diversity index and the number of observed taxa did not reveal any differences in alpha diversity among samples grouped by surgery × iron supplementation, weight, or surgery × weight. We subsequently assessed sample dissimilarity using the Aitchison distance. Once again, the ordination of samples using Principal Component Analysis (PCA) and hierarchical clustering based on the calculated distance suggests that surgery, iron supplementation, and increased weight do not drive any gross changes in the gut microbial composition. We statistically validated our observations using PERMANOVA and obtained no significant results (Supplementary Table S1). Analysis of variance suggested that females who underwent surgery had a different microbiome composition than the other females (*F* = 4.579; *p*-value = 0.041); however, the observed difference could be due to heterogeneity in variance between the two groups (Supplementary Table S1).

**Figure 2.**
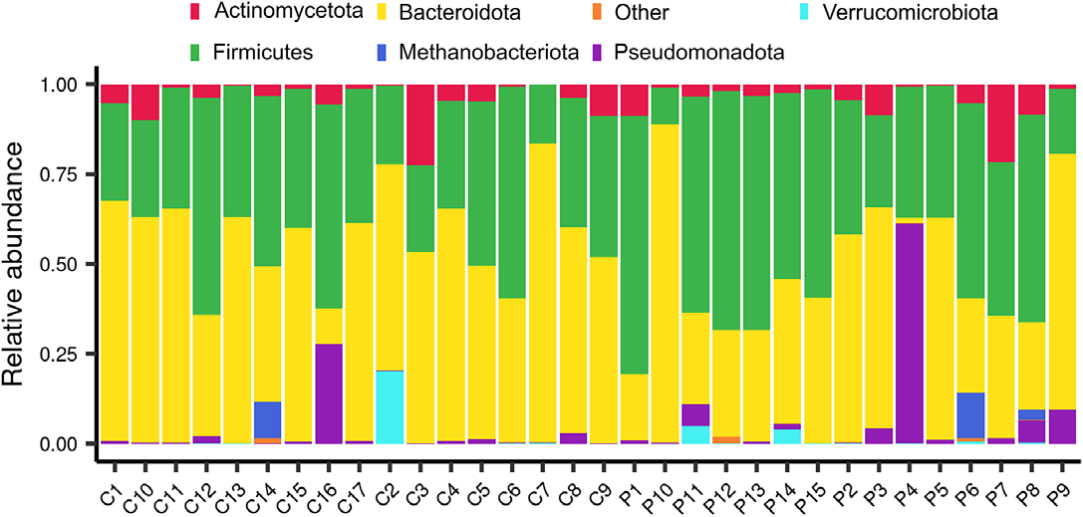
The relative abundance of the most common bacterial phyla in samples from bariatric surgery patients (P) and control subjects (C). The stacked bar plots illustrate the number of each type of microbe present in all the samples, with each bar representing a single sample. The samples are sorted by ID, with the letters “P” and “C” at the beginning of the ID numbers indicating that the person had undergone bariatric surgery and the other person did not, respectively.

**Figure 3.**
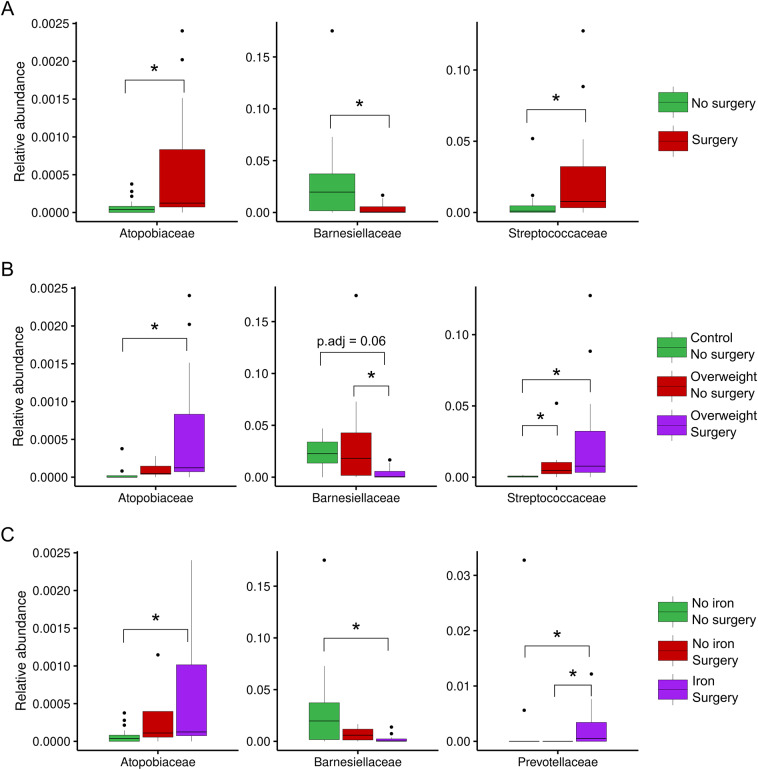
Relative abundance of important bacterial families in different clinical subgroups. (A) Abundance of bacteria in individuals who had undergone bariatric surgery or not. (B) Comparison of the abundance of bacteria between individuals grouped by both surgery and body mass index. (C) Comparison of the abundance of bacteria between individuals grouped by surgery and those treated with iron supplements. Statistical significance was examined using the Wilcoxon rank-sum test for two conditions and the Kruskal–Wallis *H* test, followed by the Dunn post hoc test for multiple conditions (*adjusted *p* < 0.05).

The relative abundances of phyla and families were subsequently compared between the same sample groups. In terms of the abundance of each sample, Bacteroidota and Bacillota appeared to be the predominant phyla, except for the P4 and P6 samples, which were obtained from patients who underwent surgery, and the C16 and C2 control samples (Figure 2). In more detail, some surgical patients (such as P3, P4, and P9) presented a higher abundance of Pseudomonadota (purple) than others. Methanobacteriota and Verrucomicrobiota were not commonly observed, but they were present in some individuals (e.g., C14, C2, P5, and P7), indicating that rare phyla can vary from person to person. Some control samples, such as C16 and C2, also contained noticeable amounts of Pseudomonadota and Verrucomicrobiota, which indicated that the same environmental or dietary factors affected both groups. As expected from their fairly stable composition across samples, no differences in the relative abundance of bacterial phyla were observed for individuals grouped by surgery, surgery × weight, or surgery × iron supplementation. An examination of the relative abundance at a lower taxonomic level revealed certain changes in the bacterial families *Atopobiaceae*, *Barnesiellaceae*, *Streptococcaceae*, and *Prevotellaceae.* Specifically, the abundance of *Atopobiaceae* appeared to increase after BS (Figure 3A); however, iron supplementation may have been the driving factor behind the observed difference (Figure 3C). The number of *Barnesiellaceae* decreased after BS (Figure 3A–C), whereas the abundance of *Prevotellaceae* seemed to increase following iron supplementation (Figure 3C). Finally, the abundance of *Streptococcaceae* increased after surgery (Figure 3A); however, when body mass index was also considered, it was revealed to be the primary variable affecting the abundance of this bacterial family (Figure 3B).

### The potential function of the gut microbiota

**Figure 4.**
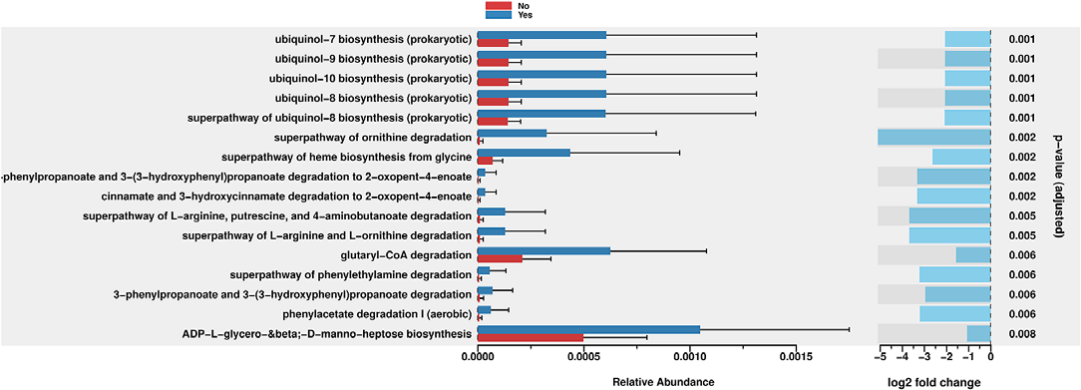
Bar plot of MetaCyc abundance data and pathways. Differentially abundant metabolic pathways between groups (Yes vs. No). Bars show relative pathway abundance with error bars indicating variability across samples. The panel on the right displays log2 fold changes for significantly enriched pathways, highlighting shifts in ubiquinone biosynthesis, amino acid metabolism, aromatic compound degradation, and select central carbon–energy pathways.

**Figure 5.**
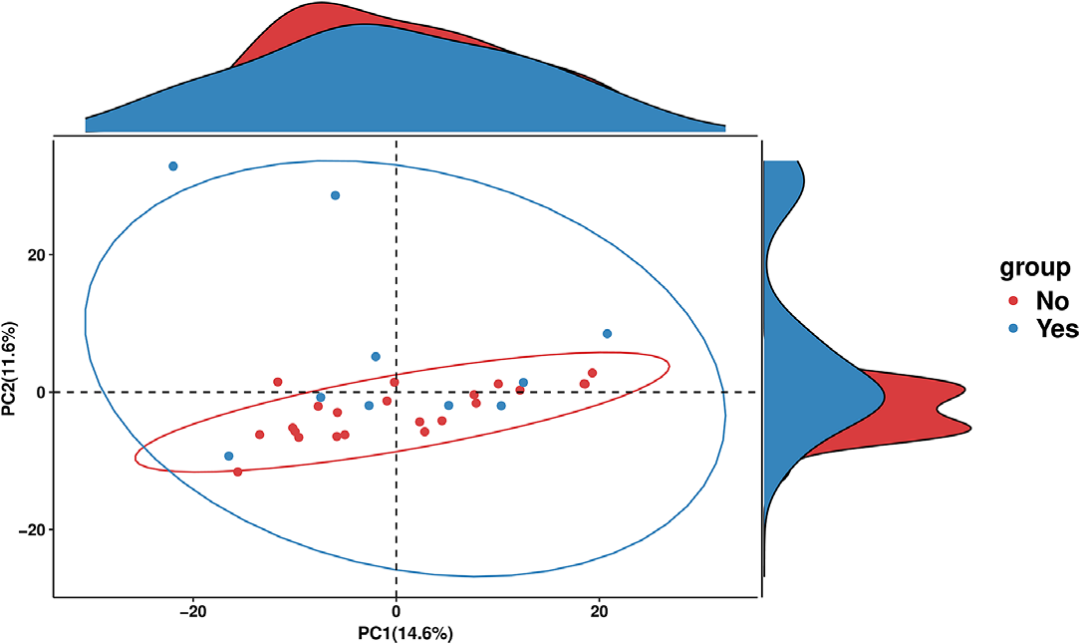
PCA plot of MetaCyc abundance data. PCA of MetaCyc pathway profiles comparing the “Yes” and “No” groups. Points represent individual samples, with 95% confidence ellipses illustrating group dispersion and overlap. Marginal density plots show how each group is distributed along the two principal components.

PICRUSt2 was used to predict the abundance of functional gene families within the intestinal microbiota of the two groups (Yes – underwent surgery) and (No – did not undergo surgery). An analysis using the MetaCyc database with the R language was performed to categorize metabolic processes, and the results indicated that the intestinal microbiota of both groups primarily engaged in metabolic activities, including biosynthesis, the superpathway of ornithine degradation, and the superpathway of haem biosynthesis from glycine (Figure 4). The Principal Coordinate Analysis (PCoA) results derived from MetaCyc metabolic pathways indicated statistically significant differences in metabolic pathways between the two groups (Figure 5). The intestinal microbiota exhibited three significantly upregulated signalling pathways, namely, PWY0–1241, PWY-5177, and PWY-5855, in the Yes group (Figure 4 and Table 2). Unfortunately, although several KEGG pathways had *p*-values < 0.05 (ko00440, ko00680, ko00562, and ko00250), their adjusted *p*-values were >0.05, indicating that none of the traits remained statistically significant after adjustment for multiple testing. The 3-HYDROXYPHENYLACETATE degradation pathway and ARGDEG pathway are involved in the degradation and production of amino acids, whereas ARG + POLYAMINE synthesis is responsible for polyamine synthesis. The three routes exhibited notable differences in the “Yes” group, as illustrated in Table 2.

**Table 2.** Differential abundance of MetaCyc pathways between individuals who underwent surgery and those who did not undergo surgery

Feature	Method	*p*-value	FDR-adjusted *p*-value	Description
3-HYDROXYPHENYLACETATE-DEGRADATION-PWY	LinDA	0.00057866	0.012832631	4-hydroxyphenylacetate degradation
ARG + POLYAMINE-SYN	LinDA	0.002278898	0.026188978	Superpathway of arginine and polyamine biosynthesis
ARGDEG-PWY	LinDA	0.00171412	0.005874743	Superpathway of L-arginine, putrescine, and 4-aminobutanoate degradation

The enrichment of pathways related to ornithine degradation and haem biosynthesis from glycine decreased in the “Yes” group, as indicated in Figure 4. The “superpathway of haem biosynthesis from glycine” differed significantly between the groups (*p* = 0.002). This pathway involves the synthesis of haem molecules, which are essential cofactors for haemoglobin, cytochromes, and other haemoproteins. This pathway was more abundant in the “Yes” group than in the “No” group. However, the negative log2 fold change indicated that, despite higher abundance, a trend towards downregulation was observed in the “Yes” group.

The bacterial degradation of ubiquinol biosynthesis pathways, PWY-5855 (ubiquinol-7, ubiquinol-8, ubiquinol-9, and ubiquinol-10 biosynthesis), seemed to be affected in the “Yes” group (Figure 4 and Table 2), all with *p*-values of 0.001, which were the lowest *p-*values in the figure. Among these equally significant pathways, when considering the magnitude of difference (considering both the relative abundance bars and the log2 fold change), the ubiquinol-7 biosynthesis pathway appeared to exhibit one of the greatest differences between groups. This pathway is involved in the production of ubiquinol-7, which is a form of coenzyme Q with a 7-unit isoprenoid side chain. While this pathway exhibited a greater absolute abundance in the “Yes” group, it showed a substantial negative log2 fold change, indicating significant downregulation in the “Yes” group compared with the “No” group when it was normalized appropriately.

PWY-5177 corresponds to glutaryl-CoA degradation. Glutaryl-CoA dehydrogenases are flavoproteins that engage with iron–sulphur clusters throughout their catalytic cycle, mainly via their interaction with electron-transferring flavoproteins. Glutaryl-CoA dehydrogenases are enzymes that catalyse the oxidative decarboxylation of glutaryl-CoA to crotonyl-CoA, thereby playing a crucial role in amino acid metabolism. They do not contain iron–sulphur clusters but transfers electrons to electron-transferring flavoproteins, which contain sulphur clusters. This cluster enables electron transfer from glutaryl-CoA dehydrogenases to the mitochondrial respiratory chain, thereby contributing to ATP production (Wischgoll et al., 2009).

Moreover, the third pathway, PWY0–1241, encodes ADP-L-glycero-β-D-manno-heptose, whose biosynthesis differs (*p* = 0.008). It is a precursor for lipopolysaccharide (LPS) biosynthesis in Gram-negative bacteria, which is crucial for their pathogenicity and the host’s immunological response. The significant variation in this pathway implies that different groups may have different populations of Gram-negative bacteria or different membrane compositions. Inflammation and host–microbe interactions may be affected. This finding indicates that while both groups use this route, the “No” group uses it far less.

Two related pathways were significantly enriched: “3-phenylpropanoate and 3-(3-hydroxyphenyl)propanoate degradation” and “3-phenylpropanoate and 3-(3-hydroxyphenyl)propanoate degradation to 2-oxopent-4-enoate.” Both showed *p*-values of 0.002–0.006, indicating significant differences between groups. These pathways are involved in the breakdown of aromatic compounds derived from plant materials. The ability to degrade these compounds is often associated with the gut microbiota, which can process plant-derived phenolic compounds. The downregulation in the “Yes” group suggested an altered capacity for processing these plant-derived compounds.

## Discussion

The gut microbiome has a significant influence on an individual’s susceptibility to illness and the effectiveness of treatments, as it affects metabolism, the immune system, and the nervous system. Certain microbial metabolites play crucial roles in these processes. The potential for customized therapeutic strategies based on the microbiome rather than providing a singular solution for all diseases has been noted in previous studies (Schupack et al., 2022). Recent studies by Brooks et al. (2023) and Hernández-Calderón et al. (2022) have revealed that age, environment, nutrition, and exercise alter the richness and variety of the microbiome, highlighting the complex factors that influence gut microbial communities. In the context of BS, failure to achieve weight loss goals and subsequent weight regain are typically linked to high-fat and high-sugar diets (Kim, 2023), with BS considered unsuccessful if weight loss is <25% after 5 years (Aminian et al., 2015). The response of the gut microbiome to interventions varies significantly; some microbial communities undergo rapid changes, whereas others maintain relative stability. Bacteroidota species may help manage obesity, but studies have shown mixed results regarding the ratio of Bacillota/Bacteroidota in healthy individuals compared with those who are obese. Our study investigated the relationships among BS, weight loss outcomes, and the gut microbiota composition, with particular attention given to the influence of iron supplementation on microbial communities.

Building on this understanding, we examined how BS, body weight, and iron supplementation interact to shape specific shifts in microbial families and their metabolic capacities. Figure 3A shows a high abundance of *Atopobiaceae*, *Barnesiellaceae*, and *Streptococcaceae* post surgery. Thus, BS might help some types of microbes grow faster. The effects of overweight and surgery status were assessed in Figure 3B, and this group had the highest prevalence of *Atopobiaceae* and *Streptococcaceae.* However, this group still seemed to have lower levels of *Barnesiellaceae.* Therefore, the host’s metabolic state may also influence how surgery alters the microbial composition, which could impact its effectiveness. Adding iron resulted in further adjustments in Figure 3C, especially in patients who had surgery and received iron. Our research, along with earlier studies by Dje Kouadio et al. (2024), indicates that taking iron supplements affects gut bacteria, leading mainly to lower levels of *Lactobacillaceae* and *Actinobacteria.* These alterations may influence the metabolic environment of the gut and potentially impact weight regulation and BS outcomes. In this study, surgery significantly increased the relative abundance of *Atopobiaceae* in the gut microbiota, particularly in overweight individuals and those receiving iron supplements. The high abundance of *Atopobium* was significantly associated with the primary products of protein fermentation, indicating its role in protein degradation within the gastrointestinal tract (Zhang et al., 2009). Moreover, the abundance of *Barnesiellaceae*, characterized as saccharolytic bacteria, was markedly elevated in the no-surgery, lean-control, and no-iron-supplementation groups, which is consistent with the findings of Del Chierico et al. (2018). This conclusion is consistent with the findings of Zhang et al. (2025), who reported a significant decrease in the abundance of *Barnesiella* in the faeces of individuals with type 2 diabetes. In addition, ALDEx2 was used to analyse DAAs for the genera retained after GCN rectification; all the genera were found to be non-abundant. No significant differences in the Bacillota/Bacteroidota ratio were observed between the surgery group and the overweight group. These findings are consistent with those of Pedroso et al. (2025), who identified gut microbiome and genetic features that predict weight loss success 12 months after sleeve gastrectomy. Individuals who achieved remission from type 2 diabetes mellitus following sleeve gastrectomy exhibited a higher abundance of Bacteroidota, regardless of the surgical method employed (Murphy et al., 2017). Despite the overall similarity in microbial diversity, we observed specific taxonomic differences that may have functional significance. We detected a higher abundance of *Streptococcaceae* in the BS group than in the non-surgical control group. These findings align with those of a study by Mabey et al. (2020), which revealed that 12 years after surgery, patients who received BS had a significantly higher prevalence of *Streptococcaceae* and a substantially lower prevalence of *Bacteroidiaceae.* These findings suggest that dietary habits are influencing factors, as *Streptococcaceae* thrive under low-pH, high-sugar conditions and metabolize sugars to produce acid (Onyango et al., 2020). Additionally, Fouladi et al. (2021) reported a significant increase in the abundance of *Streptococcus* after BS. Their cross-cohort analysis revealed that *Streptococcus* was a key taxon in the consistent postsurgical microbial signature, along with *Veillonella* and *Akkermansia.*

Moving from taxonomy to function, a comparison of the enrichment of microbial metabolic pathways between the “Yes” and “No” groups based on MetaCyc annotations showed that the ARG + POLYAMINE-SYN pathway substantially differed in the BS group, suggesting altered polyamine synthesis. This finding is significant because changes in the gut microbiome associated with metabolic syndrome and obesity-related type 2 diabetes can disrupt the production of polyamines and subsequently affect the production of substances such as spermine and spermidine, which are critical for preserving ageing and metabolic health (Bui et al., 2023). Polyamines are associated with numerous aspects of cellular function, including cell proliferation, DNA stabilization, and cellular repair. Eating probiotics, especially yogurt supplemented with *Bifidobacteria*, has been shown to increase polyamine levels in the human gut, which may improve gut health, increase lifespan, and improve overall quality of life (Lian et al., 2022). Our findings suggest that altered polyamine metabolism may represent one mechanism by which the gut microbiota influences metabolic outcomes following BS. Ubiquinol biosynthesis pathways, particularly those involved in ubiquinol-7 biosynthesis, also differed significantly between groups. Ubiquinol, the reduced form of ubiquinone (also known as coenzyme Q), plays a crucial role in the mitochondrial electron transport chain and serves as an antioxidant. A reduction in ubiquinol production may impair the ability of cells to generate energy and protect themselves from damage, potentially leading to issues with cellular function or food processing in the body (Staiano et al., 2023). Enteric iron and the gut microbiota structure are shaped by redox stress, as unbound iron is a pro-oxidant that promotes reactive oxygen species production via the Fenton reaction, potentially altering the intestinal redox balance. We hypothesize that this redox shift could favour the growth of opportunistic taxa and influence the activity of enzymes in the LPS–heptose pathway. The production of ADP-L-glycero-β-D-manno-heptose is central to LPS production in Gram-negative bacteria. Gram-negative taxa might adapt to iron-rich, oxidative conditions by upregulating this pathway. This process could lead to an increase in inflammation and the systemic translocation of cell wall components (Malesza et al., 2022). Additionally, we hypothesize that the iron-mediated redox state affects the metabolism of dietary polyphenol compounds, which are abundant in plant-based diets and modulate microbial iron absorption. Microbial degradation of aromatic polyphenols may facilitate bacterial growth by reducing iron chelation or acting as a carbon source. Iron-dependent redox enzymes can further accelerate these degradation processes. These interactions suggest that dietary modifications and iron supplementation following surgery establish a selective milieu in which iron availability and redox dynamics regulate the metabolism of plant-derived compounds and increase the stability of Gram-negative cell walls (Seyoum et al., 2021).

We acknowledge several limitations of this study. Due to volunteers’ embarrassment, acquiring stool samples was challenging, which limited our ability to statistically identify modest differences in microbial diversity across groups. Larger sample sizes are needed to identify consistent patterns in the microbiome because of substantial interindividual variability. Some methodological considerations are also important. This work utilized 16S rRNA gene sequencing to determine the taxonomic classification, but it has limited resolution for species-level identification and functional predictions. We employed bioinformatics methods to predict metabolic pathways, although a metagenomic or metabolomic analysis might provide more information on these pathways.

## Conclusions

Our findings indicate that overall microbiota diversity may be insufficient to identify nonresponders to weight loss interventions. In contrast, shifts at the family level and alterations in iron-dependent metabolic functions, such as LPS–heptose pathway activity, may provide improved discriminatory power, suggesting that these markers are particularly informative in the context of iron supplementation. The observed changes in the polyamine synthesis pathway are based on metagenomic or functional interference rather than metabolite quantification. Future studies should directly assess polyamine levels in stool and serum samples to validate the alterations in these predicted pathways and clarify their biological significance.

## Supporting information

10.1017/gmb.2026.10017.sm001 Tashkandy supplementary material Tashkandy supplementary material

## Data Availability

The accepted paired-end, primer-trimmed reads were deposited in the National Center for Biotechnology Information (NCBI) under Submission ID: SUB14906866, BioProject ID: PRJNA1193787.

## Supplementary material

The supplementary material for this article can be found at http://doi.org/10.1017/gmb.2026.10017.
